# Omphalomesenteric duct remnant adenocarcinoma in adults: a case study

**DOI:** 10.1186/s40064-016-3713-0

**Published:** 2016-11-28

**Authors:** Bingchuan Zhou, Hao Lai, Yuan Lin, Xianwei Mo

**Affiliations:** Department of Gastrointestinal Surgery, Affiliated Tumor Hospital of Guangxi Medical University, 71 Hedi Road, Nanning, 530021 Guangxi Autonomous Region China

**Keywords:** Vitelline duct, Omphalomesenteric duct remnant, Adenocarcinoma

## Abstract

**Introduction:**

The omphalomesenteric duct (OMD) or the vitelline duct (VD) is the embryonic structure connecting the vitelline sac to the primitive gut. It undergoes obliteration at 5–9 weeks of gestation. Failure of this duct to close, which occurs in approximately 2% of the population, can lead to various types of VD residual diseases. A persistent OMD remnant is pathological, and it typically presents in the pediatric population. Meckel diverticulum is the most common anomaly that results from failure of resorption of the OMD. In extremely rare instances, OMD remnant adenocarcinomas have been reported in the adult population.

**Case description:**

In this study, we present a case of OMD remnant adenocarcinoma with axillary lymph node metastases in an adult male.

**Discussion and Evaluation:**

Because OMD remnant adenocarcinoma is rare, few relevant studies have been reported. The final diagnosis of navel VD residual adenocarcinoma depends on postoperative pathology and immunohistochemical analysis. The follow-up treatment in OMD is similar to the chemotherapy regimens of postoperative gastrointestinal malignant tumors.

**Conclusions:**

In this report, the patient experienced no complications after surgery and was discharged on the seventh postoperative day, followed by 12 courses of postoperative FOLFOX6 scheme chemotherapy. By the end of chemotherapy, the patient had no evidence of recurrent disease and metastasis across the reexamination of PET–CT.

## Background

The omphalomesenteric duct (OMD) or the vitelline duct (VD) is the embryonic structure connecting the vitelline sac to the primitive gut, which undergoes obliteration at five to nine weeks of gestation (Bagade and Khanna 2015). Failure of this duct to close, which occurs in approximately 2% of the population, can produce various types of residual VD disease (Levy and Hobbs 2004). Meckel’s diverticulum is the most common congenital abnormality of residual VD disease (Khati et al. 1998), which is usually diagnosed in the pediatric population. Although a few reports have described OMD remnants in the adult population, to date, there are no reports on cancerous OMD remnants. We present the case of a 56-year-old male patient with an OMD remnant adenocarcinoma and axillary lymph node metastasis.

## Case presentation

**Fig. 1:**
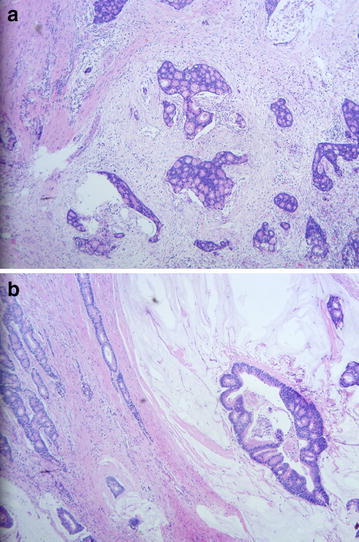
**a**, **b** Histopathological examination of the lymph nodes in the right axillary region. Hematoxylin and eosin stain (H & E stain), 40× magnification

**Fig. 2:**
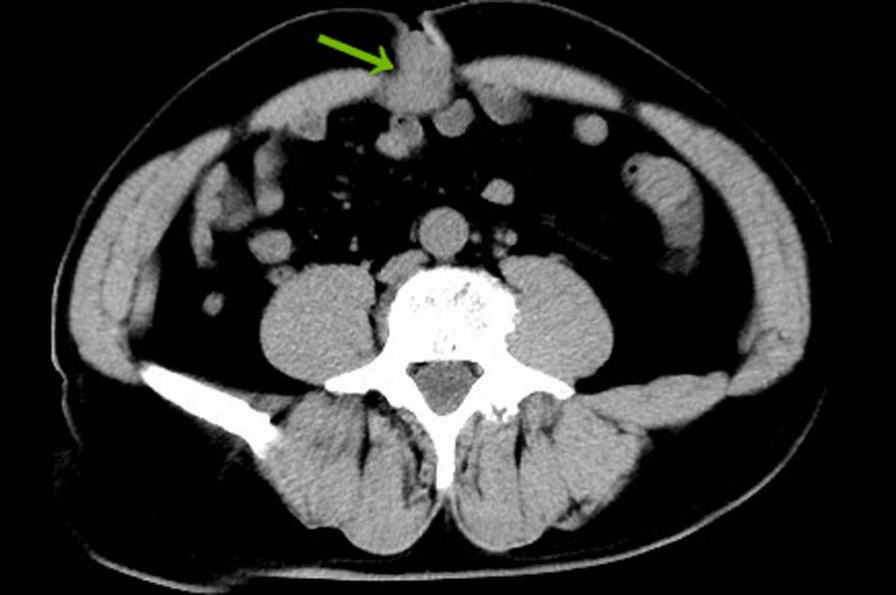
Abdominal CT scan: umbilicus lump (*arrow*), which was suspected to be an umbilicus tumor

**Fig. 3:**
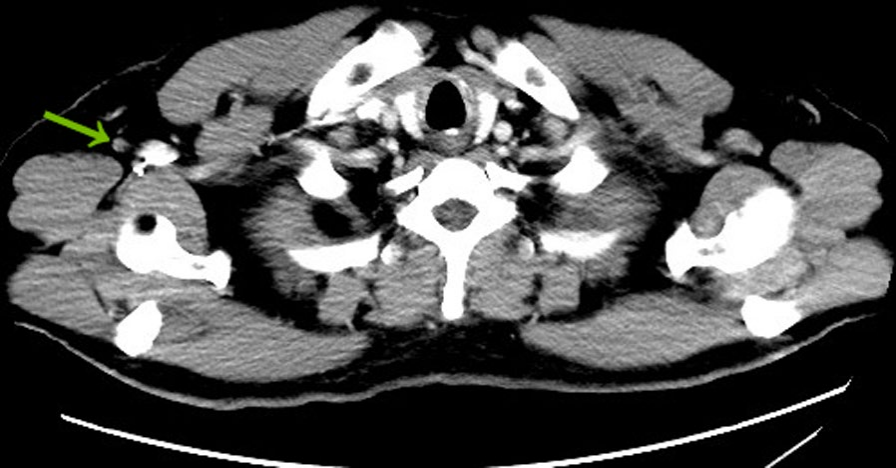
CT scan: postoperative changes of lymph nodes in the right axillary area (*arrow*). The density of soft tissue around this structure was increased

**Fig. 4:**
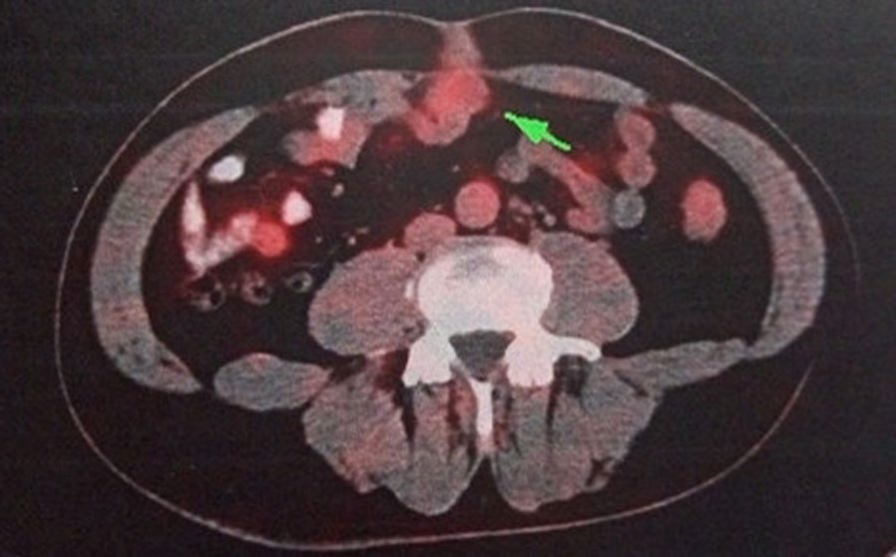
The PET–CT scan: umbilicus lump (*arrow*). An increased metabolic rate was revealed. Radioactive tracer: 18F-FDG

**Fig. 5:**
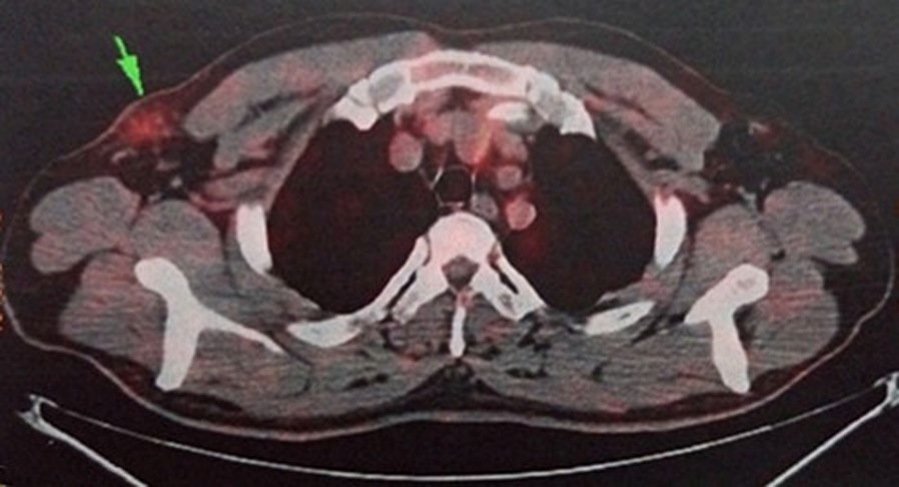
The PET–CT scan: axillary lymph nodes (*arrow*). Radioactive tracer: 18F-FDG

A 56-year-old male presented to our hospital in June 2015 with a 1-month history of an axillary lump. Preoperative pathological examination confirmed it as a metastatic adenocarcinoma through regional lymph node resection (Fig. 1). The patient denied any pain, fevers, chills, or any significant weight loss. The patient had no family history of cancer or hereditary intestinal disorders. Physical examination revealed a hard, mobile, 3 × 2 cm lesion with unclear border and a smooth surface, which was palpable at the position beneath the umbilicus. Clinical examination results for the respiratory, cardiovascular, gastrointestinal, and nervous systems and routine laboratory examination results were normal. Some tumor markers were slightly higher than the marginal values (CEA: 10.02 ng/mL, normal range: 0–6.5 ng/mL; CA72-4: 17.12 U/mL, normal range: 0–6.7 U/mL). A computed tomography (CT) scan of the abdomen confirmed the mass under the umbilicus, but it was difficult to differentiate it from the intestine. The soft tissue density around the mass was increased. The lesion measured approximately 2.8 × 2.0 cm (Fig. 2). Several lymph nodes were palpable in the right axillary area (Fig. 3). The 18F-fluorodeoxyglucose (18F-FDG) positron emission tomography**–**computed tomography (PET–CT) scan of the umbilicus mass revealed a focally increased metabolic rate, and there was no clear border line with adjacent bowel loops (Fig. 4). There was no significant uptake in the right abdomen. Increased metabolic rate was also found on the right side of axillary lymph nodes (Fig. 5), which may have been caused by postoperative inflammation. No other areas of increased metabolic uptake were identified.

**Fig. 6:**
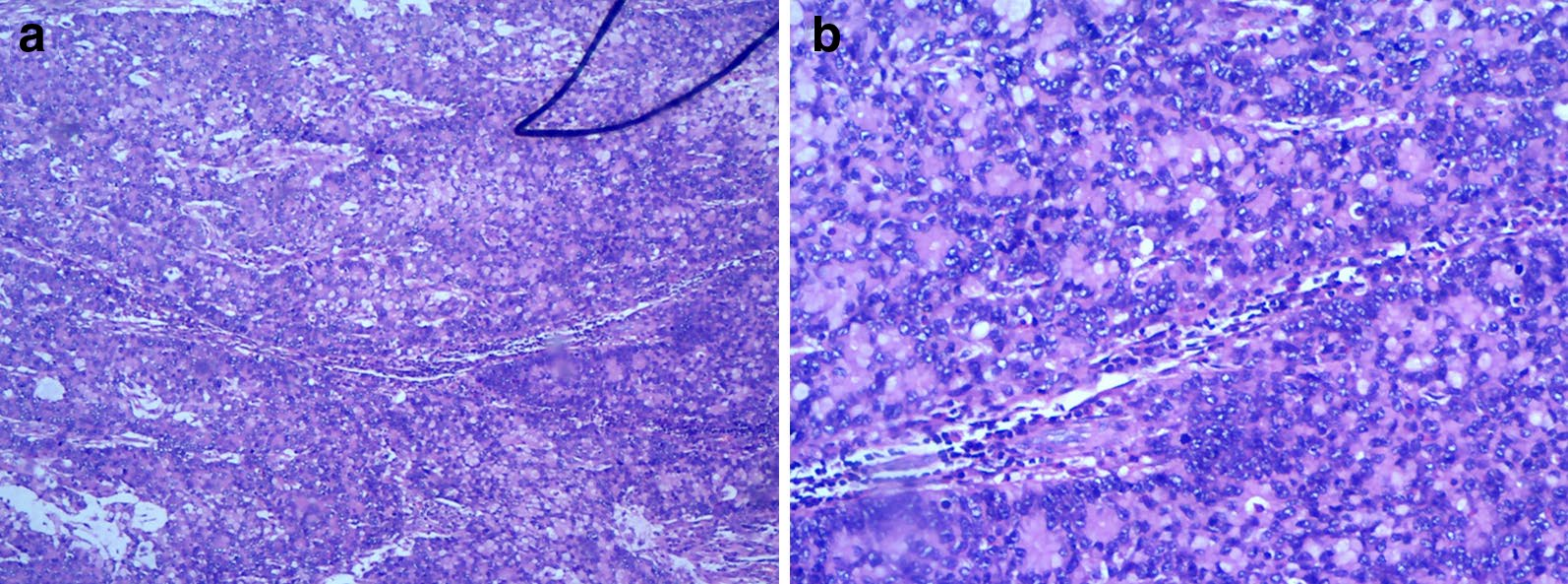
Representative images of the postoperative pathological examination of the umbilicus. H & E stain, **a** 100× magnification; **b** 400× magnification

Based on the preoperative evaluation, we first performed a laparoscopic exploration to exclude other diseases. Then, open surgery was performed to remove the bellybutton neoplasm and urachus. Postoperative pathological examination confirmed that the specimen from the transected umbilicus and urachus tissue was moderately differentiated adenocarcinoma (Fig. 6). Immunohistochemistry analysis results were as follows: CK7(+), CK20(+), Villin(+), CDX-2(±), MUC2(+), MUC5A(+), MUC6(−), TTF-1(−), PSA(−), PAX8(−), and GATA-3(−). The patient experienced no complications after surgery and was discharged on the seventh postoperative day. After 12 courses of postoperative FOLFOX6 scheme chemotherapy, the patient had no evidence of recurrent disease or metastasis across the reexamination of PET–CT.

## Discussion

Congenital gastrointestinal malformations comprise approximately 6% of all congenital anomalies (Yahchouchy et al. 2001). VD remnants are some of the most common congenital gastrointestinal anomalies and result in several other anomalies, including omphalomesenteric fistulas, enterocysts, fibrous bands connecting the intestine to the umbilicus, and Meckel’s diverticulum (Uppal et al. 2011). Nevertheless, Meckel’s diverticulum accounts for 90% of all VD anomalies. It occurs more frequently in males, and in the majority of cases, it is asymptomatic and only incidentally discovered during laparotomies conducted for other reasons, either through imaging or laparoscopy (Park et al. 2005; Bani-hani and Shatnawi 2004; Matsagas et al. 1995; Pinero et al. 2002; Sinha 2005).

Vitelline duct is seen most often in neonates and infants (Kamii et al. 1992). Very few cases have been reported in adults. In this report, we present the case of a 56-year-old male with asymptomatic OMD remnants with axillary masses. However, pathological examination proved that these were both metastatic adenocarcinoma. The pathogenesis of residual VD adenocarcinoma is not yet clear; gland metaplasia in residual gastrointestinal mucosal epithelia may be the foundation of the pathogenesis of residual VD adenocarcinoma. Axillary lymph node metastasis was probably associated with the superficial shallow lymph circumfluence of the abdominal wall in this patient’s case. Above the navel, the superficial vein drained into the axillary vein via the thoracic abdominal veins, accompanied by corresponding vein lines of lymphatic backflow into the axillary lymph nodes; below the umbilicus, lymph circumfluence was present in the shallow inguinal lymph nodes. We reviewed the literature, but no similar cases were found. Thus, we believe that this may be the first report of an OMD remnant adenocarcinoma with axillary lymph node metastasis in an adult.

The correct diagnosis of an OMD remnant before surgery is often difficult because an OMD remnant simulates many other abdominal pathologies. Differential diagnoses include Crohn’s disease, colonic diverticulitis, perforated neoplasms, pelvic inflammatory disease and urachal remnants (when localized to the periumbilical region) (Salemis 2009). A previous study presented diagnostic modalities that are used in the investigation of patients with suspected OMD remnants (Ioannidis et al. 2012). The case we report here had no typical symptomatic VD remnants. PET–CT is valuable for the diagnosis of gastrointestinal tumors. Combined PET–CT scanners have been introduced into clinical practice to provide additional information about tumors (Antoch et al. 2003). In this case, the PET–CT was the foundation of the diagnosis of the VD tumor.

In the present case, OMD remnant adenocarcinoma was suspected from the physical and imaging examination findings. Surgery provided a definitive diagnosis of an OMD remnant and curative treatment by resection with minimal invasion (Morita et al. 2015). Importantly, this procedure provided both a good field of view around the umbilicus and an adequate working space in which to definitively diagnose the OMD remnant. Although it is a rare clinical entity, a complicated OMD remnant should always be kept in mind in patients presenting with an umbilical mass.

Omphalomesenteric duct remnant adenocarcinoma is very rare, and few relevant studies have been reported. The combined pathological type, operation findings, imaging findings and even a colonoscopy are needed for diagnosis. Final diagnosis of the navel VD residual adenocarcinoma is made on the basis of postoperative pathological and immunohistochemical results; follow-up treatment should choose one type of chemotherapy regimen of postoperative gastrointestinal malignant tumors.

Patients with axillary lymph node metastases require adjuvant chemotherapy 6 months after resection of the primary tumors (Des Guetz et al. 2010). This therapy might include FOLFOX (Maindrault-goebel et al. 2000), CapeOX (Schmoll et al. 2007), or FLOX (Kuebler et al. 2007). For patients who cannot use oxaliplatin, the single drug capecitabine (Twelves et al. 2005) or 5-FU/LV (Haller et al. 2005) can be used. FOLFOX is among the most effective regimens for treating resistant gastrointestinal cancer (Maindrault-goebel et al. 2000). FOLFOX6 chemotherapy was administered to this patient. A clinical review of this patient’s treatment revealed an ideal curative effect and no signs of recurrence or metastasis.

## Conclusion

We have presented an extremely rare case of OMD remnant adenocarcinoma safely resected by surgery in an adult patient. Imaging plays a critical role in the diagnosis of OMD remnants. An awareness of the variety of these anomalies is essential to their proper management.
